# Quality Formation Mechanism of Stiff Silkworm, *Bombyx batryticatus* Using UPLC-Q-TOF-MS-Based Metabolomics

**DOI:** 10.3390/molecules24203780

**Published:** 2019-10-21

**Authors:** Dongxu Xing, Guanwang Shen, Qingrong Li, Yang Xiao, Qiong Yang, Qingyou Xia

**Affiliations:** 1 Sericulture and Agri-Food Research Institute, Guangdong Academy of Agricultural Sciences, Guangzhou 510610, China; dongxuxing@126.com (D.X.); xiaoyang@gdaas.cn (Y.X.); 2 Biological Science Research Centre of Southwest University, Chongqing 400716, China; gwshen@swu.edu.cn

**Keywords:** *Bombyx batryticatus*, UPLC-Q/TOF-MS-based metabolomics, stiff time, anticonvulsant effects, quality formation mechanism

## Abstract

*Bombyx batryticatus* is a well-known animal in traditional Chinese medicine. The aim of the research was to reveal the quality formation mechanism of *B. batryticatus* and to screen out the characteristic component used for the quality control. The anticonvulsant effects of *B. batryticatus* with a stiff time of one, five, and nine days (D1, D5 and D9, respectively) and healthy silkworm of the same developmental stage (SW) were determined by animal experiment. The dynamic changes in chemical composition were analyzed using UPLC-Q-TOF-MS-based metabolomics. D5 and D9 *B. batryticatus* exhibited significant anticonvulsant effects (*p* < 0.05 and *p* < 0.01, respectively). Accordingly, principal component analysis (PCA) and partial least squares discrimination analysis (PLS-DA) indicated that the chemical composition of D5 and D9 *B. batryticatus* changed significantly. The different metabolites mainly consisted of primary metabolites such as lipids and amino acids and secondary metabolites such as flavonoids, beauvericin, and glycolipids. Interestingly, the relative abundance of quercetin-7-*O*-β-d-4-*O*-methylglucoside, the characteristic component of *B. batryticatus*, increased with stiff time and was promised to be used as an index component of quality control. The results expand our understanding of the quality formation mechanism of *B. batryticatus*. In addition, it highlights the potential of UPLC-Q-TOF-MS-based metabolomics for the quality control purpose of TCMs.

## 1. Introduction

*Bombyx batryticatus* is a well-known animal in traditional Chinese medicine (TCM), which is the dried larva of *Bombyx mori* infected by *Beauveria bassiana* (Bals.) Vuill [1]. In traditional medical applications in China, South Korea, and Japan, *B. batryticatus* has the functions of relieving spasm by calming endogenous wind, dispelling pathogenic wind for relieving pain, dissipating phlegm, and resolving masses. It is used to treat liver wind with phlegm, convulsion, acute panic in children, tetanus, stroke, fever, headache, sore throat, itchy rubella, as well as mumps [2,3,4,5]. Modern pharmacological investigations have proved that water or alcohol extracts of *B. batryticatus* have anticonvulsant, antiepileptic, anticoagulant, hypoglycemic, antioxidant, and antitumor activities [6,7,8,9]. The demand for *B. batryticatus* is expected to increase annually with developments in pharmacological research and its application in medicine, food, and cosmetics.

Currently, *B. batryticatus* is mainly derived from silkworms spontaneously infected by *B. bassiana* in sericulture. Thus, the supply of *B. batryticatus* varies each year, and the quality is uneven [10]. However, due to the lack of efficient production technology and quality control systems, the artificial production of *B. batryticatus* has not started yet. The fundamental cause for the situation lies in an unclear quality formation mechanism of *B. batryticatus*.

**Figure 1:**
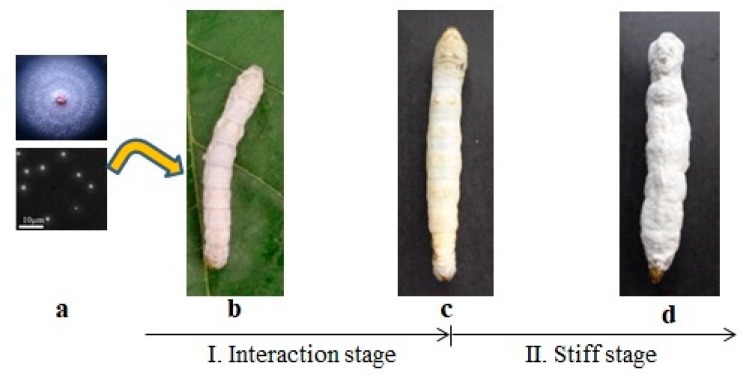
Formation process of *B. batryticatus*. (**a**) *Beauveria bassiana* spores; (**b**) silkworm larva; (**c**) death of silkworm infected by *Beauveria bassiana*; (**d**) *B. batryticatus*.

The formation process of *B. batryticatus* is shown in Figure 1. *B. bassiana* spores (Figure 1a) adhere to the body wall of the silkworm (Figure 1b) and invade its body, eventually causing death of the silkworm (Figure 1c). The process is the interaction stage of *B. mori* and *B. bassiana*. After death, *B. bassiana* grows and propagates rapidly by absorbing the silkworm nutrients. The silkworm body gradually hardens and the body surface is covered with white spores (Figure 1d) [11]. This process is called the stiff stage [12].

Many studies have reported that fungi play an important role in the transformation of active components of TCMs, and the original components can be transformed into new components with higher activity by fungal fermentation [13,14,15]. *B. bassiana* can transform >300 kinds of substrates by hydroxylation, redox reaction, sulfonation, and hydrolysis of epoxide 4-*O*-methylglucoside [16,17]. Previous studies have shown that the interaction stage seems unconnected to the quality formation of *B. batryticatus* [18,19]. The stiff stage may be a crucial period in the quality formation of *B. batryticatus*. However, few relative studies have been reported.

Epilepsy is a major neurological disorder characterized by recurrent, unprovoked seizures, which brings great pain to patients [20]. About one-third of cases are resistant to drug treatment [21]. Thus, the identification of new anticonvulsant molecules is necessary. Anticonvulsion is the main pharmacological activity of *B. batryticatus*. In this study, we compared the anticonvulsant effects of *B. batryticatus* at different stiff time in the strychnine-induced epileptic model in mice. The metabolic characteristics of the stiff stage were characterized by metabolomics, and the dynamic changes in chemical composition of *B. batryticatus* were studied. The characteristic components reflecting the quality of *B. batryticatus* were screened, which help us to understand the quality formation mechanism of *B. batryticatus* and provide important information for the technical optimization of standardized production and quality control of *B. batryticatus*.

## 2. Results

### 2.1. Changes in B. batryticatus during Stiff Stage

**Figure 2:**
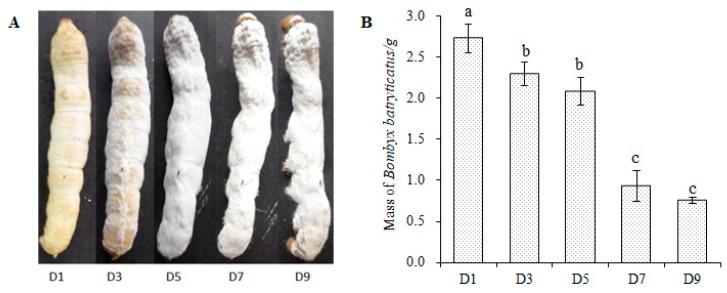
Changes in *B. batryticatus* during the stiff stage. (**A**) Appearance changes. D1, D3, D5 and D9 indicate 1, 3, 5 and 9 days after *B. batryticatus* death; (**B**) mass changes. The statistical data were treated by ANOVA and Student-Newman-Keuls (S-N-K) test. Different letters refer to the significant differences (*p* < 0.05).

The change in appearance of *B. batryticatus* is shown in Figure 2A. When the silkworm had just died, the head and chest protruded forward, and the body was soft and slightly elastic (D1). Then the corpse gradually hardened. The white airborne hyphae were exposed to the body surface on D3. Five days later, the airborne hyphae had spread all over the body. Seven days later, the silkworm body had become dry and the body mass decreased significantly (Figure 2B). Major changes took place during the stiff stage and D1, D5 and D9 silkworms were used for the following experiments.

### 2.2. Effect of Stiff Time of B. batryticatus on Anticonvulsant Efficacy

The anticonvulsant efficacy of aqueous extracts of the samples (SW, D1, D5, and D9) was compared. The dosage of crude drugs was 25 g/kg. The results showed that the efficacy of *B. batryticatus* was closely correlated with stiff time (Table 1). Compared with the model group, the D5 group had significantly prolonged clonic seizure latency (*p* < 0.05). The D9 group had prolonged clonic seizure latency (*p* < 0.01) and tonic seizure latency (*p* < 0.05), and improved mortality protection rate. The results indicated that the longer stiff time and the higher degree of stiffness, the better medicinal quality of *B. batryticatus*.

### 2.3. Metabolic Profile of B. batryticatus during Stiff Stage

**Figure 3:**
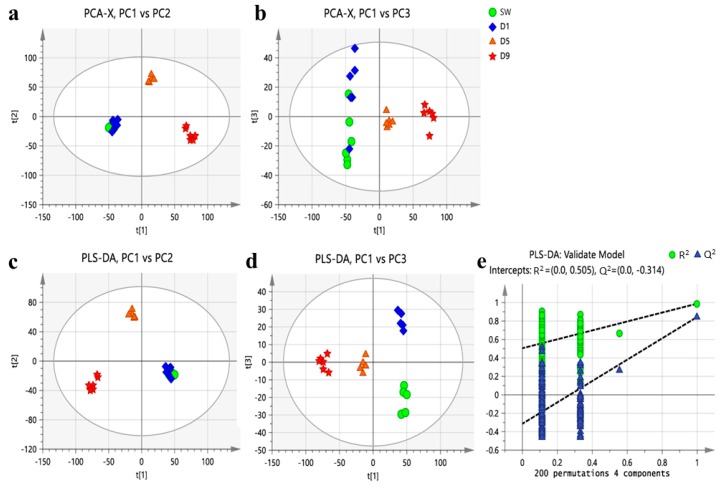
Scores plot of the principal component analysis (PCA) and partial least squares discrimination analysis (PLS-DA) of *B. batryticatus* from different stiff time. (**a**) PCA score plot (PC1 vs. PC2); (**b**) PCA score plot (PC1 vs. PC3); (**c**) PLS-DA score plot (PC1 vs. PC2); (**d**) PLS-DA score plot (PC1 vs. PC3); (**e**) presentation of chance permutation at 200 times used for the discrimination between the four groups [R^2^ = (0.0, 0.505), Q^2^ = (0.0, −0.314)].

The base peak intensity of the ion flow diagram of representative *B. batryticatus* samples is shown in Supplementary Figure S1. Principal component analysis (PCA) and partial least squares discrimination analysis (PLS-DA) were carried out for *B. batryticatus* from different stiff time (D1, D5, and D9) and healthy silkworm samples (SW) (Figure 3). In the PCA score plots, each symbol in the diagram represented a sample, and the same symbol represented the same stiff time. It can be seen that the samples with the same stiff time were clustered in similar spatial positions, indicating that their metabolic equilibrium was similar. The sample symbols of different stiff times were completely separated, which showed that the metabolic balance of *B. batryticatus* was constantly changing with the increase in stiff time. The healthy silkworms (SW) were located in the same area as those of D1, which indicates that the metabolite composition of these two groups of samples was similar.

PLS-DA is a supervised statistical method of discriminant analysis. Therefore, compared with the PCA model, it has a better classification effect. The permutation test (*n* = 200) showed that the PLS-DA model was stable and repeatable. In accordance with the results of PCA, the four groups of samples were divided into three clusters: SW and D1 samples, D5 samples, and D9 samples. The changes in metabolic balance of *B. batryticatus* were visualized by three clusters, which clearly indicated that the changes in chemical composition of *B. batryticatus* mainly occurred in the stiff stage.

### 2.4. Analysis of Different Metabolites of B. batryticatus from Different Stiff Time

According to SW vs. D1, D1 vs. D5, and D5 vs. D9, two-to-two comparisons were made, and the differential metabolites between the groups were screened. Thirty-two differential metabolites were identified as shown in Supplementary Table S1, and 35 lipids were obtained by comparing with lipid theory library (Supplementary Table S2). Compared to the SW group, twenty-seven differential metabolites were identified in the D1 group. Twenty-two metabolites were significantly decreased and only five chemical components were significantly increased (Table 2). Many flavonoids decreased dramatically. In addition, the content of amino acids such as L-glutamine, L-asparagine, and L-arginine also decreased significantly, and the content of betaine, acetylcarnitine, uracil, and hypoxanthine increased remarkably.

After 5 days of the stiff process, the chemical constituents of *B. batryticatus* changed significantly (Table 3). Forty-three differential metabolites were obtained; twenty-one were significantly lower and twenty-two were significantly higher than in the D1 group. Among them, the lipid metabolism was active, and the content of many diglycerides (DG), monoacylglycerols (MG) and unsaturated fatty acids (UFA) changed significantly. The transformation of the flavonoids was more obvious. Quercetin-7-*O*-β-d-4-*O*-methylglucoside, kaempferol-7-*O*-β-d-4-*O*-methylglucoside, and an unknown flavonoid increased significantly, while kaempferol and another unknown flavonoid decreased. In addition, the content of beauvericin, the metabolite of *B. bassiana*, also increased sharply.

Compared with the D5 group, fifty-three differential metabolites were obtained in the D9 group, including twenty-eight that decreased and twenty-five that increased (Table 4). The changed composition was mainly comprised of lipids and amino acids. The content of triglyceride (TG) decreased sharply, the content of DG, MG and phosphatidylcholine (PC) and lysophosphatidylcholine (LPC) increased significantly. In addition, the amino acids such as L-leucine, L-isoleucine and L-phenylalanine also decreased significantly. Besides, the content of quercetin-7-*O*-β-d-4-*O*-methylglucoside, kaempferol-7-*O*-β-d-4-*O*-methylglucoside, and two unknown flavonoids increased.

### 2.5. Changes in Chemical Constituents during the Stiff Stage of B. batryticatus

**Figure 4:**
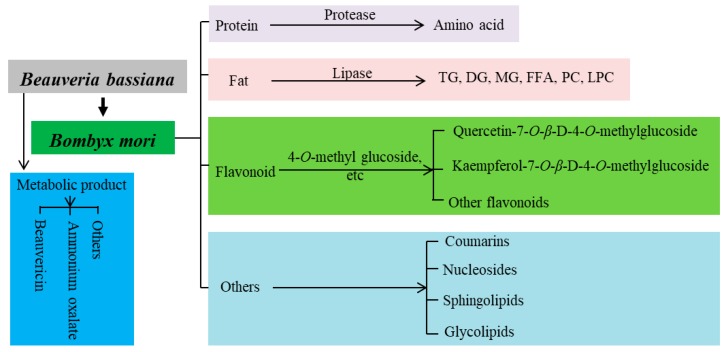
Changes in chemical constituents of *B. batryticatus* during the stiff stage.

Based on the above analysis of metabolites, the changes in metabolites of *B. batryticatus* during the stiff stage were mainly concentrated on flavonoids, amino acids, and lipids (Figure 4). Compared with the SW group, the varieties and contents of flavonoids in the D1 group changed obviously. Compared with the D1 group, the concentrations of quercetin-7-*O*-β-d-4-*O*-methylglucoside and kaempferol-7-*O*-β-d-4-*O*-methylglucoside in the D5 group were significantly increased. In addition, the silkworm fat was decomposed with the aid of the lipase secreted by *B. bassiana*. Compared with the D5 group, the concentration of TG in the D9 group decreased significantly, and the concentrations of DG, MG, PC, and LPC increased significantly. Moreover, the silkworm protein was also broken down into amino acids. The fatty acids and amino acids were used for the growth and propagation of *B. bassiana*. Also, the types and concentrations of coumarins, nucleosides, organic acids, and sphingolipids also changed greatly. For example, the concentrations of betaine and L-carnitine increased, the concentration of hypoxanthine decreased, and the concentrations of acetylcarnitine and sphingosine tended to increase first and then decrease. *B. bassiana* also secretes beauvericin and other secondary metabolites. The concentration of beauvericin in the D5 group was significantly higher than in the D1 group. However, there was no significant difference between the D5 and D9 groups.

### 2.6. Screening of B. batryticatus Quality Control Index Component

**Figure 5:**
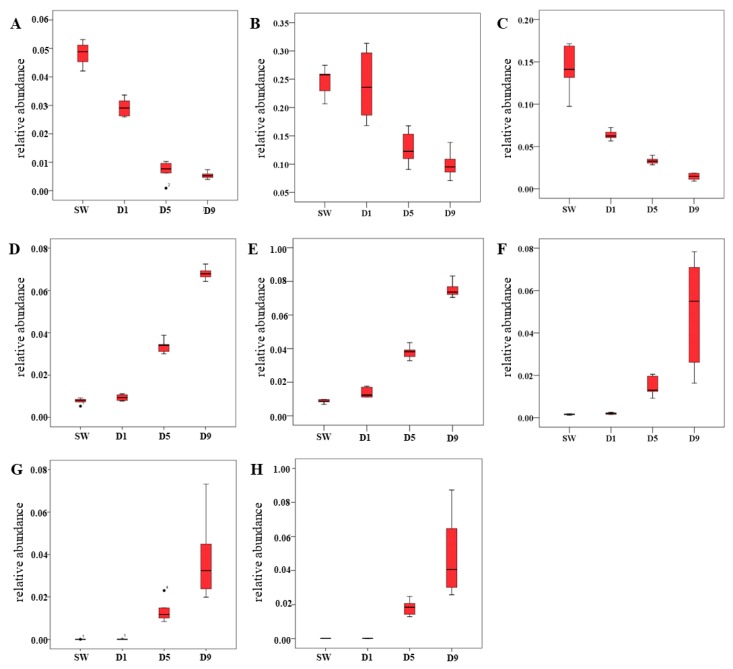
Relative abundance of eight chemical constituents. (**A**) L-Histidine; (**B**) choline; (**C**) 2-pyrrolidone-5-carboxylic acid; (**D**) L-carnitine; (**E**) betaine; (**F**) 6-hydroxynicotinic acid; (**G**) quercetin-7-*O*-β-d-4-*O*-methylglucoside; (**H**) kaempferol-7-*O*-β-d-4-*O*-methylglucoside.

By analyzing the dynamic changes in chemical composition of *B. batryticatus* during the stiff stage, eight chemical constituents related to the degree of stiffness were identified, and the relative abundance showed a tendency to decrease or increase with stiff time (Figure 5). Based on the animal experiments, *B. batryticatus* with >5 days stiff time had a marked anticonvulsant effect, and showed better medicinal quality. Therefore, the index component should be able to distinguish D5 and D1 *B. batryticatus* effectively. According to VIP > 1.5, FC > 2 and *p* < 0.001, quercetin-7-*O*-β-d-4-*O*-methylglucoside was obtained. The relative abundance of quercetin-7-*O*-β-d-4-*O*-methylglucoside was little in the SW and D1 groups but higher in the D5 and D9 groups, which indicated that it is a characteristic compound of the stiff stage of *B. batryticatus*. Furthermore, the relative abundance of quercetin-7-*O*-β-d-4-*O*-methylglucoside increased with stiff time. Therefore, quercetin-7-*O*-β-d-4-*O*-methylglucoside is promised to be an index component for quality control of *B. batryticatus*.

## 3. Discussion

*B. batryticatus* has been used as a traditional medicine for many centuries in China based on its good pharmacological activities. The anticonvulsant activity of *B. batryticatus* is very remarkable [11]. Thus, the quality formation mechanism and quality control of *B. batryticatus* in the paper was discussed mainly focusing on its anticonvulsant activity. The anticonvulsant effects of *B. batryticatus* (D1, D5, and D9) and healthy silkworm (SW) were compared in animal experiments. The results showed that the anticonvulsant effect of *B. batryticatus* was obviously related to the stiff time, which was in accordance with UPLC-Q-TOF-MS analysis. PCA and PLS-DA indicated that the change in chemical composition of *B. batryticatus* was significant during the stiff stage. Meanwhile, the metabolite composition of the SW and D1 groups was closely related. It proved that the stiff stage had an important influence on the formation of pharmacological components of *B. batryticatus*.

According to the literatures, the total flavonoid extracts have significant anticonvulsant effects. During the stiff process of *B. batryticatus*, the types and concentrations of flavonoids changed significantly. Compared with the D1 group, the concentrations of quercetin-7-*O*-β-d-4-*O*-methylglucoside and kaempferol-7-*O*-β-d-4-*O*-methylglucoside increased significantly in the D5 and D9 groups. It was reported that *B. bassiana* can react 4-*O*-methylglucoside with phenols as substrate [22]. *B. bassiana* could transform quercetin into quercetin-7-*O*-β-d-4-*O*-methylglucoside [23]. Kaempferol-7-*O*-β-d-4-*O*-methylglucoside could also be obtained by co-culture of *B. bassiana* and kaempferol [24]. Thus, it was speculated that 4-*O*-methylglucoside reaction was also an important way of flavonoid biotransformation during the stiff process of *B. batryticatus*. By the reaction quercetin and kaempferol in the silkworm body were transformed into quercetin-7-*O*-β-d-4-*O*-methylglucoside and kaempferol-7-*O*-β-d-4-*O*-methylglucoside. The flavonoid constituents specific to *B. batryticatus* probably were related to its anticonvulsant effects. However, since the compounds have not yet been isolated, we have not tested them separately to analyze their overall contribution to the anticonvulsive properties. It will be our next focus of the study content.

Moreover, ammonium oxalate and beauvericin, the metabolites of *B. bassiana*, were also known active components of the anticonvulsant effects of *B. batryticatus* [25]. According to our previous research, the content of ammonium oxalate increased with the stiff time. The metabonomics analysis showed that the beauvericin increased markedly in the D5 group and remained relatively stable in the D9 group. The results corresponded basically to the animal experiment.

In addition to its anticonvulsant effect, *B. batryticatus* has other pharmacological activities [26]. For example, polypeptide of *B. batryticatus* has obvious anticoagulant effect [7,27]. However, its route of synthesis is still unknown. In the present study, the types and concentrations of amino acids of *B. batryticatus* changed obviously during the stiff process. Also, the lipid metabolism was also active. Compared with the D5 group, the concentration of TG in the D9 group decreased significantly, and the concentrations of DG, MG, PC, and LPC increased significantly. This inferred that the protein and fat of *B. mori* were hydrolyzed into amino acids and free fatty acids by protease and lipase of *B. bassiana*. During this process, the active polypeptides of *B. batryticatus* were formed.

Besides, the type and relative concentration of coumarins, nucleosides, organic acids and glycolipids also changed greatly during the stiff stage. For instance, the relative concentration of betaine and L-carnitine increased with the stiff time, sphingosine increased first and then decreased. Pharmacological studies have shown that betaine has obvious sedative, hepatoprotective, and antitumor effects [28]. L-carnitine can reduce weight and improve abdominal obesity, dyslipidemia, and other symptoms of metabolic syndrome [29]. Sphingosine can promote the formation of ceramide in lipid cuticles, which plays an important role in moisturizing skin, thus delaying aging, which may be related to the whitening and freckling of *B. batryticatus*. The results enrich our understanding of the active components and pharmacological effects of *B. batryticatus*.

As an animal drug containing complicated compounds, quality evaluation and control of *B. batryticatus* remains challenging for modern researchers. The existing quality standard of *B. batryticatus* is inadequate to identify and grade it in the market [30]. Therefore, there is an urgent need to establish a scientific standard for the quality of *B. batryticatus*. Beauvericin and ammonium oxalate were recommended as index components for the quality control of *B. batryticatus* [31]. However, they neither reflect the complex biological transformation process of *B. bassiana* and *B. mori*, nor are specific to *B. batryticatus*. As mentioned above, quercetin-7-*O*-β-d-4-*O*-methylglucoside was the characteristic component of *B. batryticatus*. Thus, it could be used for the authenticity identification of *B. batryticatus*. Moreover, it was also an ideal marker reflecting stiff time. The anticonvulsant effect of *B. batryticatus* was significantly related to the stiff time. Thus, it was promised to be an index component for quality control of *B. batryticatus*.

## 4. Materials and Methods

### 4.1. B. batryticatus

*B. batryticatus* was derived from silkworm artificially infected by *B. bassiana*. The silkworm variety was No. 2 Liangguang. The spore suspension (1 × 10^6^/mL) of *B. bassiana* LD (Genbank ID: KM205065) was sprayed onto newly exuviated larvae of the 5th instar. Five days later the silkworms died and were placed in a clean box for further stiffening [12]. The appearance changes of *B. batryticatus* were observed on the 1st, 3rd, 5th, 7th, and 9th day (D1, D3, D5, D7, and D9, respectively) of the stiff stage. In addition, the mass of *B. batryticatus* was also weighed and compared. Each group consists of three repetitions and 100 *B. batryticatus* in each repetition. D1, D5, and D9 *B. batryticatus* was collected for the following experiments. Meanwhile, healthy silkworms at the same developmental stage were collected as controls (SW). For the metabonomics experiment, six biological repeats were taken at each time point (each silkworm was an independent biological repeat). For the animal experiment, 500 *B. batryticatus* were taken at each time point.

### 4.2. Sample Preparation

For the metabonomics experiment, the samples were immediately frozen in liquid nitrogen and then homogenated. Each sample (100 ± 5 mg) was added to 1 mL of 80% methanol and treated with ultrasound for 5 min in an ice water bath, then the supernatant was collected by freezing centrifugation (16,000 *g*, 4 °C; Eppendorf 5430R, Eppendorf, Hamburg, Germany) for 15 min. The residue was extracted again. The test sample comprised of two combined supernatants. For the animal experiment, after drying and crushing, the samples were extracted with hot water for 1 h. The residue was extracted again. Then the extract solution was concentrated, freeze-dried and the aqueous extract was obtained.

### 4.3. Animal Experiment

Male SPF Kunming mice, 18–22 g, were fed adaptively for 3 days. The mice were randomly divided into six groups: control, model, SW, D1, D5, and D9, with 10 mice in each group. Intragastric administration was done once daily for 6 days according to the preset dose (crude drugs 25 g/kg), while the control and model groups were given aseptic water of equal volume. On the 6th day, strychnine (1.6 mg/kg) was administered intraperitoneally 30 min after intragastric administration, except in the control group. The trial was approved by the Experimental Animal Ethics Committee of Sericulture and Agri-Food Research Institute, Guangdong Academy of Agricultural Sciences (June 2017, approval number: A2017-06-02).

Mice were closely observed for 30 min, and the clonic seizure latency (grade I–III seizure time) and the tonic seizure latency (grade V attack time) were recorded. The data were analyzed by SPSS version 18.0 software (IBM, Armonk, NY, USA). The measurement data were expressed as mean ± standard error (X ± SE). One-way analysis of variance (ANOVA) followed by the least significant difference (LSD) test were used to compare the data between groups. The mouse convulsion response was classified according to the Racine standard: class 0: no reaction; class I: rhythmic oral angle, ear or facial muscle clonic clonus; class II: nodding, accompanied by more severe facial muscle twitch clonus; class III: forelimb clonus but not vertical clonus; class IV: forelimb clonus with vertical clonus; class V: generalized tonic-clonic seizure and fall [32]. The number of dead mice within 30 min was counted, and the death protection rates were calculated and compared using the Chi-square test.

### 4.4. UPLC-Q/TOF-MS

Chromatographic separation was performed on a Waters ACQUITY UPLC (Waters, Milford, MA, USA) system with a Waters Acquity UPLC BEH C18 (2.1 mm × 100 mm, 1.7 μm) maintained at 50 °C. The mobile phases consisted of water (phase A) and acetonitrile (phase B), both with 0.1% formic acid (*v*/*v*). A linear gradient elution was performed with the following program: 0–0.5 min, 2% B; 1.5 min, 40% B; 6.5 min, 80% B; 9.5 min, 100% B; 13 min, 100% B; 13.4 min, 2% B and held to 15.5 min. The flow rate was 0.38 mL/min. The injection volume was 3 μL.

Mass spectral determination was performed on a Q-Tof Premier mass spectrometer (Waters) in ESI + mode with a high resolution with 0.3 s survey scan time and a range of 50–1000 *m*/*z* in the centroid mode. The capillary voltage was set to 3.0 kV. The sampling cone voltage and cone gas flow were 35 V and 50 L/h, respectively. The desolvation gas was maintained at a flow rate of 600 L/h and a temperature of 350 °C. The ion source temperature was 115 °C. To improve the identification of unknown metabolites, the MS^E^ (mass spectrometry^Elevated Energy^) function was also performed to obtain fragment ion information with a ramp collision energy from 20 to 45 eV. The mass accuracy calibration was performed with the lock mass, leucine-enkephalin at *m*/*z* 556.2771, with the data acquisition frequency set at 15 s. The software for instrument control and data collection was Masslynx version 4.1 (Waters). To monitor the stability of the metabolomic experiments, quality control samples, pooled from all samples, were prepared and analyzed with the same procedure as those of the experimental samples during whole determination.

### 4.5. Data Processing, Statistical Analysis and Metabolite Identification

The raw data of UPLC-QTOF-MS were firstly transformed to NetCDF format by DataBridge in MassLynx (version 4.1), and then processed by XCMS and CAMERA packages in the R software platform. In the XCMS package, the peak picking (method = centWave, ppm = 15, peakwidth = c (5,20), snthresh = 10), alignment (bw = 10 and 5 for the first and second grouping, respectively), and retention time correction (method = obiwarp) were conducted. In the CAMERA package, the annotations of isotope peak, adducts, and fragments were performed with default parameters. The final data were exported as a peak table file, including observations (sample name), variables (rt_mz), and peak abundances. The data were normalized against total peak abundances in Microsoft Excel (Microsoft, Redmond, WA, USA) before performing univariate and multivariate statistics.

The normalized data were imported to SIMCA software (version 14.1, Umetrics, Umeå, Sweden), where the data were preprocessed by Pareto scaling and mean centering before performing principal component analysis (PCA) and partial least squares discrimination analysis (PLS-DA). The model quality was described by the R^2^X or R^2^Y and Q^2^ values. R^2^X (for PCA) or R^2^Y (for PLS-DA), defined as the proportion of variance in the data explained by the models, indicated the goodness-of-fit. Q^2^, defined as the proportion of variance in the data predictable by the model, indicated the predictability of the current model, calculated by a cross-validation procedure. A default seven-round cross-validation in SIMCA software was performed throughout to determine the optimal number of principal components in order to avoid model over-fitting. The permutation test was also applied to evaluate the validity of the model.

For univariate analysis, the normalized data were analyzed in the R platform, where a parametric test was performed on the data in normal distribution by Welch’s *t* test. A nonparametric test was performed on the data in abnormal distribution by the Wilcoxon Mann-Whitney test. The variables with variable importance in projection (VIP) values of PLS-DA model > 1.0 and *p*-values of univariate statistical analysis <0.05 were identified as potential differential metabolites. They were identified based on their mass spectral data using the Metlin database, relevant published literature, and confirmed based on their retention times and fragmentation patterns. Fold change (FC) was calculated as the binary logarithm of average normalized peak intensity ratio between group 1 and group 2, where the positive value means that the average mass response of group 1 is higher than group 2.

## 5. Conclusions

In this study, the important role of the stiff stage in the quality formation of *B. batryticatus* was confirmed; the dynamic changes in the chemical components of *B. batryticatus* during the stiff stage were analyzed; and the quality formation mechanism of *B. batryticatus* was roughly presented. The results expand our understanding of the pharmacological activities and quality formation mechanism of *B. batryticatus*. Moreover, it provides us important information about the technical optimization of standardized production and quality control of *B. batryticatus*. In addition, UPLC-Q-TOF-MS-based metabolomics offers an excellent holistic method for the quality control of TCMs.

## Figures and Tables

**Table 1:** Efficacy of *B. batryticatus* samples on strychnine-induced epileptic seizures in mice.

Groups	Clonic Seizure Latency/s	Tonic Seizure Latency/s	Mortality Protection Rate/%
Control	ns	ns	100
Model	163.00 ± 12.60	223.90 ± 26.57	10
SW	177.4 ± 19.29	356.6 ± 160.63	20
D1	187.70 ± 19.78	387.10 ± 157.66	20
D5	222.10 ± 16.93 *	435.80 ± 152.97	30
D9	280.30 ± 21.86 **	684.60 ± 188.78 *	40

ANOVA and least significant difference (LSD) tests are used to compare the clonic seizure latency and tonic seizure latency, and Chi-square test is used to compare the mortality protection rate between groups. * *p* < 0.05 and ** *p* < 0.01 as compared to the model group. (*p* < 0.01).

**Table 2:** Differential metabolites between groups (healthy silkworm samples (SW) vs. different stiff time sample D1).

Classification	Metabolites	VIP Value	*p*-Value	FC(SW/D1)
Flavonoids	Unknown flavonoid	1.93	3.51 × 10^−3^	−6.06
	Rutin	1.13	6.01 × 10^−3^	−5.86
	Unknown flavonoid	1.8	1.63 × 10^−2^	−4.51
	Quercetin	1.68	4.04 × 10^−2^	−1.08
	Kaempferol	1.79	3.58 × 10^−2^	0.92
Amino acid	L-glutamine	4.71	1.01 × 10^−4^	−2.62
	L-asparagine	1.18	3.10 × 10^−3^	−2.37
	L-arginine	2.1	5.87 × 10^−3^	−2.14
	L-tryptophan	1.95	4.20 × 10^−3^	−1.02
	L-histidine	3.12	3.17 × 10^−6^	−0.72
	L-proline	4.45	5.95 × 10^−3^	−0.58
Lipids	TG (56:8)	8.76	1.66 × 10^−3^	−1.94
	TG (52:5)	6.83	1.21 × 10^−2^	−1.28
	TG (54:6)	7.25	6.51 × 10^−3^	−1.24
	TG (54:8)	5.23	3.54 × 10^−2^	−1.17
	TG (56:7)	9.91	1.89 × 10^−2^	−1.11
	TG (52:4)	7.99	2.10 × 10^−3^	−0.98
	PC (36:2)	0.73	1.38 × 10^−3^	−0.96
Nucleosides	Adenine	3.25	3.06 × 10^−2^	−1.05
	dTDP	1.37	8.34 × 10^−4^	−0.94
	Uracil	2.45	1.64 × 10^−4^	1.26
	Hypoxanthine	6.23	1.11 × 10^−2^	2.09
Others	Benzoic acid	1.56	2.88 × 10^−2^	−1.39
	2-Hydroxycinnamic acid	1.73	3.08 × 10^−2^	−1.37
	2-pyrrolidone-5-carboxylic acid	5.79	2.84 × 10^−4^	−1.02
	Betaine	4.53	3.37 × 10^−3^	0.65
	Acetylcarnitine	1.76	4.71 × 10^−3^	0.66

**Table 3:** Differential metabolites between groups (D1 vs. D5).

Classification	Metabolites	VIP Value	*p*-Value	FC(D1/D5)
Flavonoids	Kaempferol	1.34	5.31 × 10^−3^	−1.82
	Unknown flavonoid	1.12	4.26 × 10^−2^	−1.25
	Unknown flavonoid	1.07	3.08 × 10^−4^	4.41
	Kaempferol-7-*O*-β-d-4-*O*-methylglucoside	1.39	8.75 × 10^−5^	6.75
	Quercetin-7-*O*-β-d-4-*O*-methylglucoside	1.67	1.22 × 10^−6^	6.82
Amino acid	L-histidine	1.81	3.67 × 10^−8^	−1.8
	L-phenylalanine	3.04	2.59 × 10^−3^	0.57
	L-isoleucine	1.5	2.61 × 10^−2^	0.82
	L-valine	1.79	1.51 × 10^−5^	0.84
	L-tryptophan	1.39	6.71 × 10^−6^	1.27
	L-proline	5.38	1.03 × 10^−6^	3.22
Lipids	DG (34:3)	3.2	3.81 × 10^−4^	−2.51
	DG (36:6)	5.96	9.74 × 10^−4^	−2.27
	DG (36:4)	5.67	5.92 × 10^−4^	−1.82
	DG (34:1)	2.69	4.10 × 10^−4^	−1.58
	DG (36:5)	2.49	3.61 × 10^−3^	−1.34
	DG (36:3)	2.22	1.72 × 10^−3^	−1.1
	DG (36:2)	2.85	1.65 × 10^−3^	−1.01
	DG (33:4)	1.96	6.75 × 10^−3^	1.91
	DG (35:5)	2.3	8.92 × 10^−6^	3.37
	DG (35:6)	1.94	4.41 × 10^−6^	3.43
	MG (18:2)	1.67	3.00 × 10^−2^	−0.95
	Eicosatrienoic acid (C20:3)	1.93	1.71 × 10^−3^	−0.93
	Octadecapentaenoic acid (C18:5)	4.89	3.03 × 10^−2^	−3.26
	Octadecatrienoic acid (C18:3)	5.32	9.96 × 10^−4^	−0.68
	Octadecenoic acid (C18:1)	2.98	4.21 × 10^−2^	−0.62
	Stearic acid (C18:0)	1.96	3.51 × 10^−3^	−2.28
	Octadecatetraenoic acid (C18:4)	3.43	1.31 × 10^−6^	3.9
	Octadecadienoic acid (C18:2)	1.85	1.10 × 10^−4^	4.33
	(4*E*,2*S*,3*R*)-2-*N*-octadecanoyl- 4-tetradecasphingenine	1.63	1.93 × 10^−4^	−1.53
	(4*E*,6*E*,2*S*,3*R*)-2-*N*-eicosanoyl- 4,6-tetradecasphingadienine	1.46	3.05 × 10^−3^	−1.07
	Phytosphingosine	3.76	3.41 × 10^−5^	4.64
Nucleosides	Hypoxanthine	3.59	7.35 × 10^−3^	−2.48
	Adenine	3.41	2.57 × 10^−3^	2.05
Others	2-pyrrolidone-5-carboxylic acid	2.27	3.23 × 10^−4^	−1.09
	Choline	3.76	2.54 × 10^−3^	−0.91
	Acetylcarnitine	1.79	1.80 × 10^−5^	1.03
	Betaine	6.16	1.79 × 10^−7^	1.48
	Benzoic acid	1.23	9.91 × 10^−4^	1.83
	L-carnitine	1.97	6.95 × 10^−9^	1.84
	2-Hydroxycinnamic acid	1.43	1.10 × 10^−3^	1.9
	6-Hydroxynicotinic acid	1.36	4.38 × 10^−5^	2.86
	Beauverin	10.84	2.04 × 10^−5^	9.36

**Table 4:** Differential metabolites between groups (D5 vs. D9).

Classification	Metabolites	VIP Value	*p*-Value	FC(D5/D9)
Flavonoids	Unknown flavonoid	1.2	3.05 × 10^−2^	1.39
	Quercetin-7-*O*-β-d-4-*O*-methylglucoside	1.85	1.38 × 10^−2^	1.4
	Kaempferol--7-*O*-β-d-4-*O*-methylglucoside	1.64	1.68 × 10^−2^	1.47
	Unknown flavonoid	0.25	9.55 × 10^−4^	1.51
Amino acid	L-leucine	2.49	7.59 × 10^−5^	−3.75
	L-isoleucine	2.66	1.64 × 10^−5^	−3.43
	L-phenylalanine	5.61	1.11 × 10^−7^	−2.94
	L-tryptophan	1.64	1.95 × 10^−8^	−2.01
	L-histidine	0.62	3.54 × 10^−3^	−0.64
	L-valine	1.56	6.66 × 10^−4^	0.44
Lipid	TG (54:8)	1.68	1.35 × 10^−2^	−3.46
	TG (52:6)	3.62	4.51 × 10^−3^	−3.73
	TG (52:4)	3.15	2.75 × 10^−3^	−3.64
	TG (56:7)	3.56	3.18 × 10^−3^	−3.43
	TG (52:5)	1.89	5.91 × 10^−3^	−3.22
	TG (54:6)	2.03	4.74 × 10^−3^	−2.75
	TG (56:8)	1.64	5.63 × 10^−3^	−2.53
	DG (35:5)	1.8	9.32 × 10^−4^	−1.35
	DG (35:6)	1.17	7.87 × 10^−3^	−0.79
	DG (33:4)	1.5	5.56 × 10^−3^	−0.76
	DG (36:6)	2.91	6.60 × 10^−3^	0.99
	DG (36:2)	3.95	2.45 × 10^−5^	1.4
	DG (34:1)	2.6	1.87 × 10^−5^	1.42
	DG (34:3)	2.96	3.16 × 10^−6^	2.17
	DG (36:4)	7.5	2.69 × 10^−7^	2.24
	DG (36:3)	4.48	1.01 × 10^−6^	2.25
	DG (36:5)	4.48	2.10 × 10^−5^	2.32
	MG (22:6)	1.79	1.60 × 10^−2^	0.44
	MG (18:1)	3.84	1.54 × 10^−2^	1.82
	MG (20:3)	1.95	8.64 × 10^−5^	1.89
	MG (18:2)	6	3.01 × 10^−5^	3.17
	Octadecadienoic acid (C18:2)	1.84	1.45 × 10^−4^	−3.63
	Octadecatetraenoic acid (C18:4)	3.3	4.34 × 10^−6^	−2.71
	Eicosatetraenoic acid (C20:4)	1.4	3.33 × 10^−2^	−0.2
	LPC (14:1)	1.46	2.92 × 10^−3^	3.19
	LPC (18:1)	2.53	2.84 × 10^−6^	3.2
	LPC (18:2)	3.2	7.82 × 10^−9^	5.17
	PC (36:2)	3.07	2.06 × 10^−6^	6.2
	PC (36:4)	4.35	3.20 × 10^−7^	6.69
	PC (36:5)	3.98	1.85 × 10^−5^	7.04
	(4*E*,6*E*,2*S*,3*R*)-2-*N*-eicosanoyl- 4,6-tetradecasphingadienine	1.23	7.35 × 10^−5^	−1.48
	Phytosphingosine	2.75	2.02 × 10^−3^	−1.22
Nucleosides	Sphinganine	1.36	1.23 × 10^−2^	−1.12
	Xanthine	2.45	2.83 × 10^−3^	−4.5
	Hypoxanthine	1.43	2.58 × 10^−2^	−2.38
	Adenine	3.32	3.28 × 10^−3^	−1.76
	Acetylcarnitine	2.48	4.63 × 10^−9^	−3.23
Others	2-Hydroxycinnamic acid	1.51	1.97 × 10^−4^	−1.95
	Benzoic acid	1.32	8.65 × 10^−5^	−1.96
	2-pyrrolidone-5-carboxylic acid	1.7	9.20 × 10^−6^	−1.19
	Betaine	7.79	2.43 × 10^−8^	0.99
	L-carnitine	2.4	3.99 × 10^−8^	1.03
	6-Hydroxynicotinic acid	2.1	5.97 × 10^−3^	1.78

## Supplementary Materials

The following are available online at https://www.mdpi.com/1420-3049/24/20/3780/s1, Figure S1: Representative base peak intensity chromatograms of healthy silkworm SW (**a**), D1 *Bombyx batryticatus* (**b**), D5 *B. batryticatus* (**c**) and D9 *B. batryticatus* (**d**), Table S1: Differential metabolites identified based on VIP > 1, *p*-value < 0.05, Table S2: Mass spectrometric data of lipids in *Bombyx batryticatus*.
