# Supplementary material for: Quality Formation Mechanism of Stiff Silkworm, Bombyx batryticatus Using UPLC-Q-TOF-MS-Based Metabolomics
Source: Molecules. 2019 Oct 21;24(20):3780. doi: 10.3390/molecules24203780 (PMC6832393; doi:10.3390/molecules24203780)
Supplement: Supplementary file 1 [file molecules-24-03780-s001.zip › molecules-607571-supplementary/supplementary files v2/Table S1,2.pdf]

Table S1 Differential metabolites identified based on VIP > 1, $p < 0.05$

No.	Metabolite identification	Formula	Rt/min	m/z	MS/MS fragment ion (m/z)
1[a]	L-Histidine	$C_6H_9N_3O_2$	0.58	156.0769	110.0689, 83.0588, 56.0488, 93.0431, 82.0511, 81.0437, 95.0585, 66.0330, 54.0328, 68.0485
2[a]	L-Asparagine	$C_4H_8N_2O_3$	0.62	133.0607	74.025, 43.0189, 44.0508, 70.0297, 46.0302, 31.9901, 87.0558
3[a]	L-Carnitine	$C_7H_{15}NO_3$	0.62	162.1124	60.0791, 85.0265, 43.0166, 102.0892, 103.0365, 162.1105, 57.0317, 58.0631, 59.0715
4[a]	L-Glutamine	$C_5H_{10}N_2O_3$	0.63	147.075	84.0434, 56.0489, 41.0382
5[a]	L-Leucine	$C_6H_{13}NO_2$	1.21	132.102	44.0506, 43.0550, 41.0394, 86.0969, 30.0348, 39.0238, 69.0706
6[a]	L-Isoleucine	$C_6H_{16}NO_2$	1.57	132.1019	44.0503, 69.0704, 86.0971, 41.0395, 30.0346, 56.0504, 57.0581
7[a]	L-Phenylalanine	$C_9H_{11}NO_2$	1.82	166.0864	77.0381, 103.0534, 120.0798, 91.0534, 79.0536, 53.0388, 65.0384
8[a]	L-Tryptophan	$C_{11}H_{12}N_2O_2$	2.03	205.0977	118.0639, 115.0527, 91.0533, 143.0713, 146.0581, 117.0563, 132.0792, 130.0636, 77.0369
9[a]	L-Arginine	$C_6H_{14}N_4O_2$	0.59	175.12	70.0695, 60.0570, 43.0309, 71.0498, 68.0496, 72.0815, 130.0991, 116.0718
10[a]	L-Valine	$C_5H_{11}NO_2$	0.88	118.0863	58.0716, 42.0358, 59.0765, 43.0426, 118.0888, 56.0501, 30.0348
11[a]	L-Proline	$C_5H_9NO_2$	0.67	116.0704	70.0627, 68.0470, 43.0520
12[a]	Quercetin	$C_{15}H_{10}O_7$	2.7	303.0508	303.0519, 153.0188, 229.0509, 137.0238, 201.0554, 257.0456, 165.0189, 285.0412
13[a]	Kaempferol	$C_{15}H_{10}O_6$	3.98	287.0559	287.0586, 153.0197, 121.0283, 165.0205
14[bc]	Rutin	$C_{27}H_{30}O_{16}$	2.63	633.1436	633.1445, 331.1, 325.0312, 324.0251, 487.0834
15[bc]	Kaempferol-7-*O*-*β*-D-4-*O*-methyl glucoside	$C_{22}H_{22}O_{11}$	3.04	463.1236	287.0585
16[bc]	Quercetin-7-*O*-*β*-D-4-*O*-methyl glucoside	$C_{22}H_{22}O_{12}$	2.7	479.1183	
17[b]	Adenine	$C_5H_5N_5$	0.67	136.0621	119.0330, 136.0580, 92.0232, 65.0129, 67.0281, 94.0378, 55.0284, 40.0177
18[b]	Xanthine	$C_5H_4N_4O_2$	0.75	153.0411	110.0344, 55.0286, 136.0137, 82.0394, 81.0075, 153.0401, 53.0131
19[b]	Hypoxanthine	$C_5H_4N_4O$	0.81	137.0463	55.0281, 137.0427, 119.0326, 110.0323, 82.0379, 94.0374, 65.0128, 67.0282, 92.0230, 40.0175
20[b]	Uracil	$C_4H_4N_2O_2$	0.7	113.0347	96.0083, 70.0294, 43.0175, 40.0188, 68.0129, 42.0341, 53.0033, 95.0243, 113.0349
21[b]	Beauverin	$C_{45}H_{57}N_3O_9$	7.9	784.4217	244.1366,262.1474, 784.4242, 134.0977, 523.2863, 623.3382,541.2966, 234.1514, 362.2001
22[b]	Betaine	$C_5H_{11}NO_2$	0.64	118.0861	58.0654, 59.0729, 43.0292, 118.0860, 55.0292, 72.0558, 30.0344
23[a]	Choline	$C_5H_{14}NO^+$	0.6	104.1063	58.0651, 60.0807, 44.0498, 45.0349, 42.034
24[a]	Acetylcarnitine	$C_9H_{17}NO_4$	0.67	204.1235	85.0297, 60.0821, 57.0348, 144.1027
25[b]	Benzoic acid	$C_7H_6O_2$	1.17	123.0447	77.0392, 79.0545, 51.0231, 44.9973, 105.0335
26[b]	2-Pyrrolidone-5-carboxylic acid	$C_5H_7NO_3$	0.63	130.05	84.0453, 56.0500, 41.0392, 44.0138, 39.0240, 130.0674
27[b]	2-Hydroxycinnamic acid	$C_9H_8O_3$	1.17	165.0551	95.0491, 77.0381, 91.0537, 119.0479, 123.0431, 103.0521

28[b]	6-Hydroxynicotinic acid	$C_6H_5NO_3$	1.7	140.0343	122.0246, 78.0346, 94.0290, 51.0235, 66.0344, 140.0347, 39.0237
29[b]	Sphinganine	$C_{18}H_{39}NO_2$	6.58	302.3057	60.0441, 254.2837, 67.0540, 81.0690, 95.0847, 284.2929, 83.0830, 109.0996, 55.0549
30[c]	Phytosphingosine	$C_{18}H_{39}NO_3$	7.11	318.3002	
31[c]	(4*E*,2*S*,3*R*)-2-*N*-octadecanoyl-4-tetradecasphingenine	$C_{32}H_{63}NO_3$	10.43	532.4712	
32[c]	(4*E*,6*E*,2*S*,3*R*)-2-*N*-eicosanoyl-4,6-tetradecasphingadienine	$C_{34}H_{65}NO_3$	10.6	558.4859	

a. Reference standard； b. Blast in the database； c. Speculation based on literature.

Table S2 Mass spectrometric data of lipids in *Bombyx Batryticatus*

No.	Metabolite identification	Rt/min	m/z
1	TG(54:8), Na^+	9.7	897.7006
2	TG(52:6), Na^+	10.84	873.6991
3	TG(52:4), Na^+	13.08	877.7303
4	TG(56:7), Na^+	11.03	899.7158
5	TG(52:5), Na^+	11.8	875.7148
6	TG(54:6), Na^+	11.98	901.731
7	TG(56:8), H^+	13.25	903.7476
8	DG(35:5)	10.28	601.4807
9	DG(35:6)	9.87	599.4655
10	DG(33:4), Na^+	9.92	597.4493
11	DG(36:6), Na^+	10.55	635.4641
12	DG(36:2), Na^+	11.52	643.5269
13	DG(34:1), Na^+	11.45	617.5121
14	DG(34:3), Na^+	10.91	613.4806
15	DG(36:4), Na^+	10.95	639.497
16	DG(36:3), Na^+	11.2	641.512
17	DG(36:5), Na^+	10.72	637.4784
18	MG(22:6), H^+	8.8	403.282
19	MG(18:1), Na^+	9.02	379.282
20	MG(20:3)	9.45	381.2976
21	MG(18:2), Na^+	8.68	377.2664
22	Octadecadienoic acid (C18:2)	8.03	281.2481
23	Octadecatetraenoic acid (C18:4)	6.49	277.2167
24	Eicosatetraenoic acid (C20:4), Na^+	9.36	327.2267
25	Eicosatrienoic acid (C20:3), Na^+	9.73	329.2434

26	Octadecapentaenoic acid (C18:5)	7.1	275.2012
27	Octadecatrienoic acid (C18:3) , Na^+	8.67	301.2131
28	Octadecenoic acid (C18:1), Na^+	9.36	305.2452
29	Stearic acid	9.72	307.261
30	LPC(14:1)	8.45	466.2932
31	LPC(18:1), Na^+	8.66	544.3378
32	LPC(18:2), H^+	8.11	520.34
33	PC(36:2), Na^+	10.99	808.5836
34	PC(36:5), Na^+	10.4	802.537
35	PC(36:4), Na^+	10.56	804.554
