# Supplementary figures and images for: Quality Formation Mechanism of Stiff Silkworm, Bombyx batryticatus Using UPLC-Q-TOF-MS-Based Metabolomics
Source: Molecules. 2019 Oct 21;24(20):3780. doi: 10.3390/molecules24203780 (PMC6832393; doi:10.3390/molecules24203780)

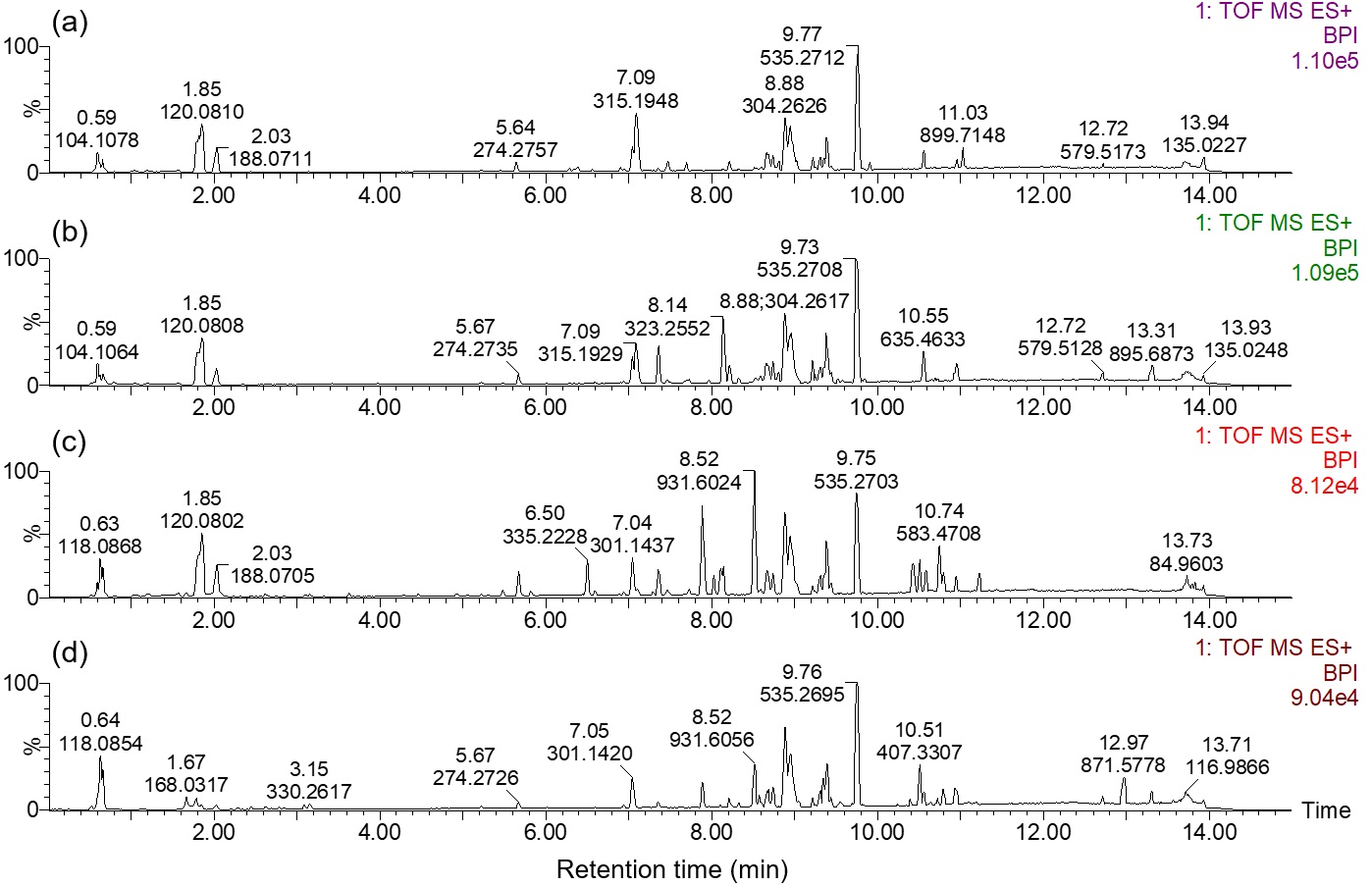

Supplement: Supplementary file 1 [file molecules-24-03780-s001.zip › molecules-607571-supplementary/supplementary files v2/Figure S1.jpg]
